# High-Entropy Spinel Ferrites with Broadband Wave Absorption Synthesized by Simple Solid-Phase Reaction

**DOI:** 10.3390/molecules28083468

**Published:** 2023-04-14

**Authors:** Xiu Chang, Zhiwei Duan, Dashuang Wang, Shushen Wang, Zhuang Lin, Ben Ma, Kaiming Wu

**Affiliations:** 1 The State Key Laboratory for Refractories and Metallurgy, Collaborative Innovation Center for Advanced Steels, International Research Institute for Steel Technology, Hubei Province Key Laboratory of Systems Science in Metallurgical Process, College of Science, Wuhan University of Science and Technology, Wuhan 430081, China; 2 College of Materials Science and Engineering, Chongqing University, Chongqing 400044, China

**Keywords:** high entropy, solid-phase reaction, spinel ferrite, porous structure, wave absorption

## Abstract

In this work, high-entropy (HE) spinel ferrites of (FeCoNiCrM)_x_O_y_ (M = Zn, Cu, and Mn) (named as HEO-Zn, HEO-Cu, and HEO-Mn, respectively) were synthesized by a simple solid-phase reaction. The as-prepared ferrite powders possess a uniform distribution of chemical components and homogeneous three-dimensional (3D) porous structures, which have a pore size ranging from tens to hundreds of nanometers. All three HE spinel ferrites exhibited ultrahigh structural thermostability at high temperatures even up to 800 °C. What is more, these spinel ferrites showed considerable minimum reflection loss (RL_min_) and significantly enhanced effective absorption bandwidth (EAB). The RL_min_ and EAB values of HEO-Zn and HEO-Mn are about −27.8 dB at 15.7 GHz, 6.8 GHz, and −25.5 dB at 12.9 GHz, 6.9 GHz, with the matched thickness of 8.6 and 9.8 mm, respectively. Especially, the RL_min_ of HEO-Cu is −27.3 dB at 13.3 GHz with a matched thickness of 9.1 mm, and the EAB reaches about 7.5 GHz (10.5–18.0 GHz), which covers almost the whole X-band range. The superior absorbing properties are mainly attributed to the dielectric energy loss involving interface polarization and dipolar polarization, the magnetic energy loss referring to eddy current and natural resonance loss, and the specific functions of 3D porous structure, indicating a potential application prospect of the HE spinel ferrites as EM absorbing materials.

## 1. Introduction 

Over the years, widespread use and rapid advancement of microwave and radio frequency communication systems have led to an intense increase in electromagnetic (EM) radiation [1]. The EM radiation strength increases at the rate of 7–14% per year, causing serious damage to human health and information security [2,3,4,5]. Many research groups have realized this problem and took a large effort to develop novel electromagnetic microwave absorbers (EMAs) to reduce EM radiation, which generally possess four basic requirements, lightweight, thin thickness, wide effective bandwidth, and high absorption efficiency [6,7,8,9,10]. Among them, wide absorbing bandwidth plays a key role in the application of EMAs, primarily due to the more and more widespread application of microwaves in various wavelengths. This requires absorbing materials to possess considerable absorption performance for each frequency EM wave [11,12,13]. Besides, environmental tolerance of EMAs has become another significant influence factor because of many extreme operating environments [14,15]. For example, the high-temperature stability of absorbing materials has attracted great attention for the application of EMAs in the field of aviation [16,17,18]. 

In general, based on the wave loss-absorbing mechanism, EMAs can be divided into two groups: dielectric loss and magnetic loss materials. The former primarily includes metal- or carbon-based materials that generally possess high electrical conductivity, and the latter mainly consisted of carbonyl iron and ferrites, which usually exhibit high magnetic conductivity. Compared with classic dielectric loss-absorbing materials (such as conductive graphite, and carbon black), ferrite materials such as EWAs have received wide attention in recent years due to their various unique crystal structures, such as spinel ferrites, garnet ferrite, and hexaferrites [19]. Among them, spinel ferrites with a general structure of AB_2_O_4_ (A: divalent ion of metal transitions; B: trivalent ion of Fe) have been extensively studied for EM wave absorption due to the inexpensive preparation procedure, high thermal stability, and excellent magnetic and dielectric loss properties [20,21,22]. For instance, Ding et al. [23,24] fabricated CuFe_2_O_4_ and Co_0.5_Cu_0.5_Fe_2_O_4_ spinel ferrites by hydrothermal method and found that the ferrites exhibit excellent microwave absorption performance, mainly attributing to the magnetic loss including eddy-current loss, magnetic resonance, and natural resonance. Liu et.al. [25] prepared the novel CNZF (Co doping in Ni–Zn ferrite)/GN (graphene) nanocomposites by a facile one-pot hydrothermal method, which performed a dual-region microwave absorption, i.e., exhibiting two strong reflection loss peaks at about 9.6 and 5.2 GHz. Zhu et al. [26] reported the NiCoO_4_ spinel ferrites with micro-nano hierarchical structures by hydrothermal synthesis. The urchin-like NiCoO_4_ exhibits the optimum reflection loss value of −41 dB and wide effective absorbing bandwidth of 15 GHz, mainly due to the unique needle-like assembly. 

Recently, high-entropy (HE) alloys as a kind of advanced materials have been extensively studied, owing to their high oxidation and corrosion resistance and excellent mechanical properties [27,28,29,30]. The HE design strategy is to form a structurally stable solid solution using multiple metal elements as the main body, which breaks the traditional alloy design method with a single element as the main body and meanwhile overcomes the disadvantages of the traditional doping technique [31]. For example, Li et al. [32] prepared the flake FeCoNi (Si_0.6_Al_0.2_B_0.2_) HE alloys magnetic powders by melt spinning and ball milling, which showed excellent microwave absorption performance. Spontaneously, this strategy was also introduced into oxide materials, known as high-entropy oxides (HEOs) [33]. Due to the cocktail effect of HE materials, HEOs exhibit better electrical and magnetic properties through the synergistic effect of various elements, resulting in the great possibility of HEOs as an ideal wave-absorbing material [34,35,36]. So far, some HE spinel ferrites have been successfully prepared. F.H. Mohammadabadi et at. [37] synthesized HE ferrite nanoparticles of (MnNiCuZn)_0.7_Co_0.3_Fe_2_O_4_ by solution combustion method. The composite of (MnNiCuZn)_0.7_Co_0.3_Fe_2_O_4_/paraffin showed the maximum reflection loss of −27 dB at the matching thickness of 5.3 mm, mainly due to the loss mechanism of interfacial relaxation and ferromagnetic resonance. The mechanism analysis reveals that the multicomponent HE ferrites exhibit more active sites and higher conductivity, owing to the multitudinous metal cations in the HE spinel structure [38]. However, the developments of novel HE spinel ferrites with excellent absorbing properties are still lacking, as well as systematic studies of their microwave absorption mechanism. 

In this work, HE spinel ferrites of (FeCoNiCrM)_x_O_y_ (M = Zn, Cu, and Mn) were prepared by a simple solid-phase reaction. Hereafter, these samples were named as HEO-Zn, HEO-Cu, and HEO-Mn for convenience, respectively. The spinel ferrite powders, with a micro-sized diameter, exhibited a three-dimensional porous structure with a pore size from tens to hundreds of nanometers. These ferrites showed excellent microwave absorbing performance and a significantly broad effective absorption band, as well as high structural thermostability. It is expected to provide an important reference for promoting the application of spinel ferrites in the field of EM wave absorption. 

## 2. Results and Discussion

### 2.1. Structural Characterization

**Figure 1:**
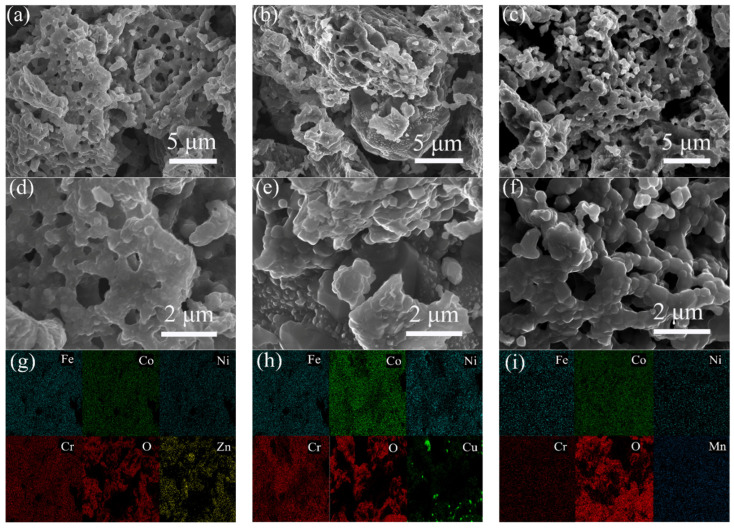
SEM images and EDS-mapping of (**a**,**d**,**g**) HEO-Zn, (**b**,**e**,**h**) HEO-Cu, and (**c**,**f**,**i**) HEO-Mn samples, respectively.

**Figure 2:**
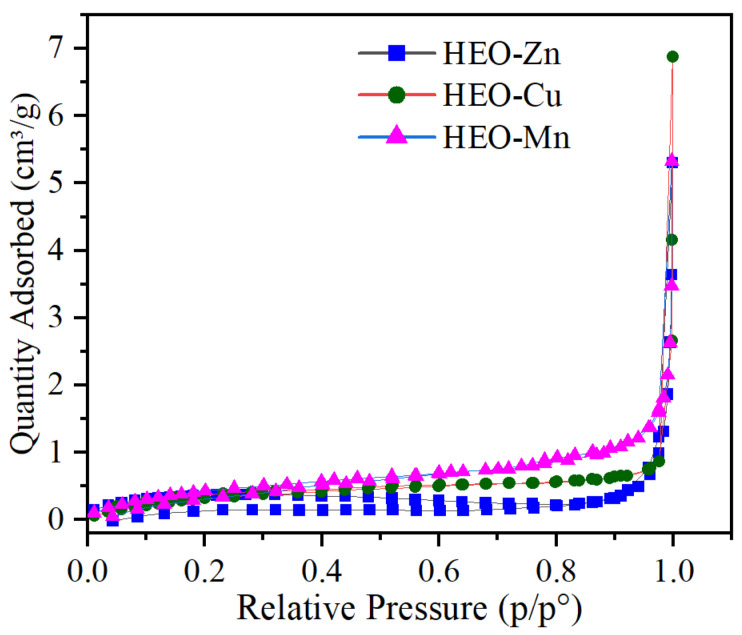
The nitrogen adsorption-desorption isotherm of HEO-Zn, HEO-Cu, and HEO-Mn.

Figure 1 shows the SEM morphologies and EDS-mapping of HEO-Zn, HEO-Cu, and HEO-Mn samples. The as-prepared samples possess a microscale powder form, and there are lots of pores among these powders, which have a diameter range from tens to hundreds of nanometers. This indicates that the homogeneous three-dimensional (3D) submicron porous structures were formed after solid phase reactions at high temperatures. Figure 2 shows the nitrogen adsorption-desorption isotherms of HEO-Zn, HEO-Cu, and HEO-Mn samples, and the specific surface areas of HEO-Zn, HEO-Cu and HEO-Mn are 1.26 m^2^/g, 1.41 m^2^/g, and 1.81 m^2^/g, respectively, indicating almost the same pore structures for the three samples. The pores were formed by mechanical uniform mixing and high temperature sintering of various raw oxides. The porous structure can not only reduce the density of wave-absorbing materials but also benefits from improving EM absorption performance. It is found from EDS-mapping in Figure 1g–i that the elements of Fe, Co, Ni, and Cr are uniformly distributed on the sample surface, as well as Zn, Cu, and Mn elements with the content being slightly different [39]. Hence, it can be concluded that the complexation solid-phase reaction was sufficiently carried out, thereby achieving the atom mixture and ultimately forming the HE ferrites. 

**Figure 3:**
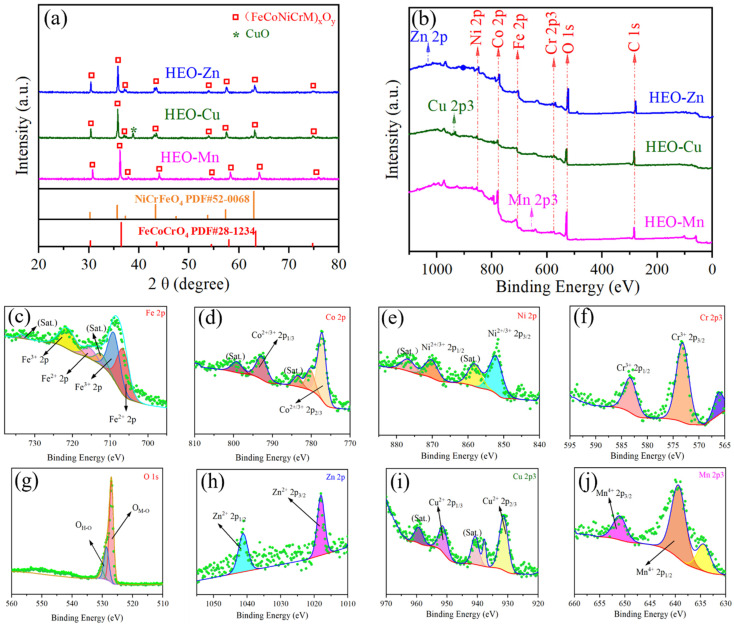
(**a**) XRD patterns, (**b**) XPS spectrums of HEO-Zn, HEO-Cu, and HEO-Mn samples. (**c**–**g**) are the XPS spectra of Fe 2p, Co 2p, Ni 2p, Cr 2p, and O 1s of HEO-Cu, and (**h**–**j**) are the XPS spectra of Zn 2p of HEO-Zn, Cu 2p of HEO-Cu, and Mn 2p of HEO-Mn, respectively.

Figure 3a shows the XRD patterns of HEO-Zn, HEO-Cu, and HEO-Mn ferrites. It is seen that all the XRD spectra are similar, and most of the peaks are relevant to those of NiCrFeO_4_ or FeCoCrO_4_ substances, manifesting the formation of a single-phase spinel structure in these HE ferrites. As we know, all the elements of Fe, Co, Ni, Cr, Zn, Cu, and Mn belong to the transition metal group and possess the approximate atomic radius, which easily forms single-phase crystal structures, as well as their oxide materials [40]. Besides, it is seen that the XRD pattern of HEO-Cu has one small peak at about 38.7 degrees, in accordance with the (111) plane of CuO, indicating the existence of residue raw substance of CuO. The residue CuO can not only increase the consecutiveness and transmission of electrons but also provide more polarized interfaces, thus benefiting the EM absorption ability of HEO-Cu. Furthermore, the crystalline grain size (*D*) can be estimated from the XRD patterns. According to the Scherrer formula, the *D* values can be expressed as:

(1) $$D=\frac{K\lambda }{\beta cos\theta }$$

where *D* is crystalline grain size, *K* is Scherrer constant, *λ* is the wavelength of the X-ray source, *β* is the full width at half maximum (FWHM), and *θ* is peak position. Thus, the grain sizes of HEO-Zn, HEO-Cu, and HEO-Mn samples can be calculated, and the results are 26.8 nm, 34.8 nm, and 43.0 nm, respectively. 

XPS experiments were also conducted to confirm the surface constitution and metal valence of these HE spinel ferrites. Figure 3b shows the full spectra of XPS patterns of HEO-Zn, HEO-Cu, and HEO-Mn samples. It is found that there are four clear and similar peaks, assigned to Fe 2p, Co 2p, Ni 2p, and Cr 2p, respectively (see Figure 3c–f). Normally, the peaks related to O were also found at the same position of 526.9 eV in all three spectra (Figure 3g). Due to the doping of different elements, these XPS spectra are of slight difference, as shown in Figure 3h–j. There are another three small peaks at about 1031.7 eV in the HEO-Zn spectrum, 936.1 eV in the HEO-Cu spectrum, and 655.5 eV in the HEO-Mn spectrum, respectively, which can be confirmed as the characteristic peaks of the Zn, Cu, and Mn elements. We can speculate that the doping elements of Zn, Cu, and Mn were successfully diffused into each spinel structure during the solid-phase reaction process, in accordance with the above EDS-mapping and XRD analyses [41]. 

**Figure 4:**
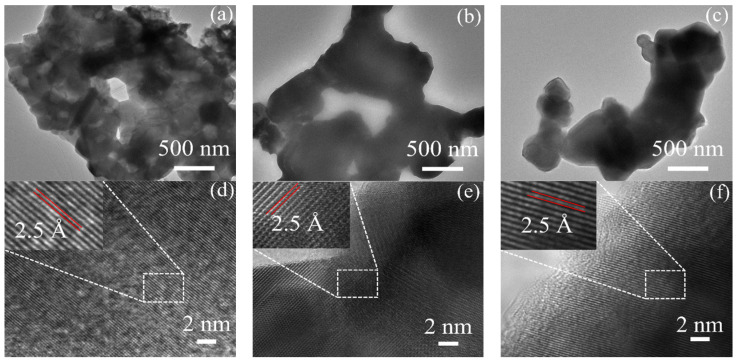
(**a**–**c**) TEM and (**d**–**f**) high-resolution TEM images of HEO-Zn, HEO-Cu, and HEO-Mn samples, respectively. Insets of (**d**–**f**) are magnified images of the relevant areas marked in (**d**–**f**).

The specific microstructures of HEO-Zn, HEO-Cu, and HEO-Mn samples were also observed by TEM, as shown in Figure 4. For all the samples, the ferrite powders are composed of spherical nanoparticles, suggesting the nanocrystalline feature of these spinel ferrites. Moreover, the submicron porous structures were also found by TEM, as shown in Figure 4a,b. Figure 4d–f shows the high-resolution TEM images of the ferrite powders selected randomly. It is seen that the lattice fringes can be clearly observed and the inter-planar distances of the three samples are all about 0.25 nm, which is equal to the inter-planar distance of (311) plane of NiCrFeO_4_ or FeCoCrO_4_ substances. This result is consistent with the XRD analysis, further proving that we have obtained the HE ferrites with a spinel structure through a solid-phase reaction. 

### 2.2. Physical Properties 

**Figure 5:**
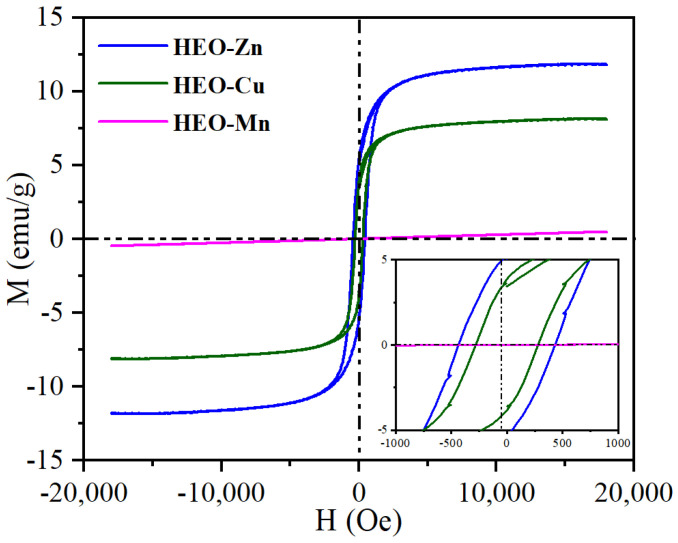
The hysteresis loops of the as-obtained samples of HEO-Zn, HEO-Cu, and HEO-Mn.

Figure 5 shows the hysteresis loops of HEO-Zn, HEO-Cu, and HEO-Mn HE spinel ferrites, recorded in the magnetic field range from −20 kOe to 20 kOe. The saturation magnetization (Ms), remanence (Mr) and coercivity (Hc) values can be obtained from the hysteresis loops, and the results are listed in Table 1. It is well known that magnetic properties are of importance for the magnetic loss of EMAs. In general, the larger Ms indicates the higher magnetic permittivity and the higher Hc might cause stronger frequency resonance, thus increasing the magnetic loss. From Figure 5, it can be seen that, except HEO-Mn, the other two samples show typical ferromagnetic properties. That is to say that Ms rapidly increases with the external magnetic field increasing and reaches a saturation condition as the external magnetic field rises to a certain value. The HEO-Zn has the highest Ms and Hc (12.0 emu/g and 433.4 Oe, respectively), whereas the decrease in magnetic properties of HEO-Mn might result from Mn being an antiferromagnetic metallic element. Therefore, it is believed that the differentiation of magnetic properties of these HE spinel ferrites is mainly due to the doping of different metallic elements and thus the changing material components [20]. 

**Figure 6:**
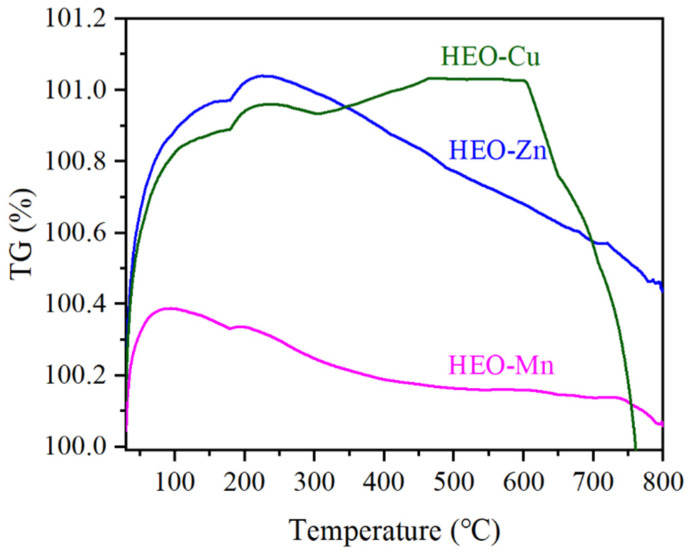
DTG curves of HEO-Zn, HEO-Cu, and HEO-Mn samples.

Figure 6 shows the DTG curves of the three ferrites of HEO-Zn, HEO-Cu, and HEO-Mn, which present the changing trend in mass with reference to temperature. It can be seen that, below 200 °C, there is a small mass increase for all the samples (the maximum ratio is about 1.0% for HEO-Zn and HEO-Cu), mainly owing to the secondary oxidation of the sample surface. Moreover, with the increase in temperature, the mass continues to decrease. Significantly, although raising the temperature up to 800 °C, the mass change is still very small (less than 1.0%). The above results indicate the ultra-high structural stability of these HE spinel ferrites at high temperatures. 

### 2.3. Wave Absorption Properties and Mechanism

**Figure 7:**
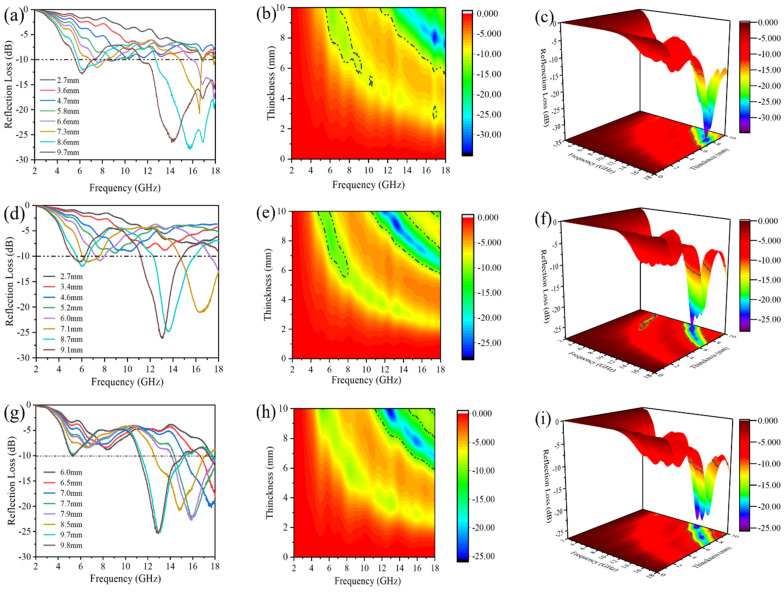
The RL curves, and 2D, 3D color-maps at different matching thickness of (**a**–**c**) HEO-Zn, (**d**–**f**) HEO-Cu, and (**g**–**i**) HEO-Mn.

Figure 7 shows the EM wave absorption properties of HEO-Zn, HEO-Cu, and HEO-Mn spinel ferrites, which were also listed in Table 2, as well as those of many reported wave absorption materials [42,43,44,45,46]. It is noteworthy that all the HE spinel ferrites in the present work possess considerable minimum reflection loss (RL_min_) and significantly enhanced effective absorption bandwidth (EAB) in comparison with the other wave absorption materials. The RL_min_ of HEO-Zn is −27.9 dB at about 15.7 GHz with a matched thickness of 8.6 mm, and the EAB is about 6.8 GHz (11.2–18.0 GHz). Whereas the HEO-Cu sample exhibits an RLmin value of −27.3 dB at about 13.3 GHz with a matched thickness of 9.1 mm, and notably, its EAB reaches about 7.5 GHz (10.5–18.0 GHz) that covers almost the whole X-band range. The RL_min_ and EAB values of HEO-Mn are −25.5 dB and 6.9 GHz, respectively. The relatively low RL_min_ in HEO-Mn is possibly due to its obviously poor magnetic properties (see Figure 5). Although the matched thickness of HEO-Cu is relatively thick, the obvious broad bandwidth can certify its fine practical application prospect in the EM wave absorption field. Basically, the results above indicate that the EAB values of these ferrites are evidently enhanced (see Table 2), which is an important performance characteristic of HEO materials [38]. It is found from the structural characterization that the morphology and pore structure of the present samples are similar, i.e., the homogeneous 3D porous structures. This porous structure can provide the 3D conductive network and abundant sample-air interfaces, and besides that the entered electromagnetic waves can perform multiple reflections in the pores, thus promoting the absorption and loss of electromagnetic waves. Moreover, for each transition metal element, it is believed that there would be the best RL_min_ at the different matched frequencies. Thereby, the synergistic action of multitudinous metal components introduced by HE effects could probably broaden the EAB property. 

**Figure 8:**
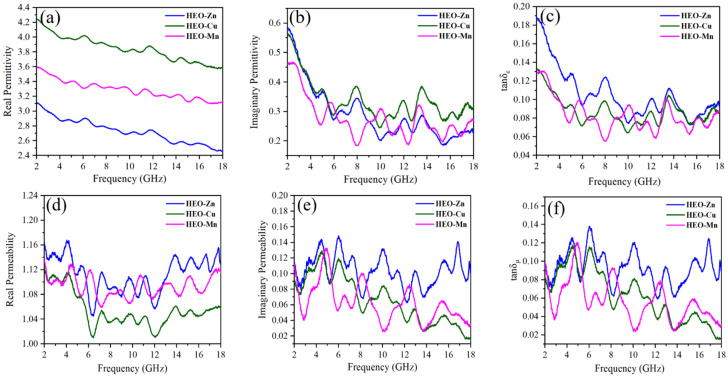
(**a**) The real part (*ε*′), (**b**) imaginary part (*ε*″) of permittivity, (**c**) dielectric loss tangent (tan*δ*_ε_), and (**d**) the real part (*µ*′), (**e**) imaginary part (*µ*″) of permeability, (**f**) magnetic loss tangent (tan*δ*_µ_) of HEO-Zn, HEO-Cu, and HEO-Mn samples.

The EM wave absorption properties are intensely related to the dielectric and magnetic loss behaviors of EMAs, which will be analyzed in detail as follows. Figure 8a,b shows the complex dielectric constants of these composites of ferrite/paraffin in the range of 2–18 GHz. The real part of permittivity (ε′) of these samples continues to decline with frequency increasing, which is attributed to the dissipative behavior of frequency caused by the hysteresis phenomenon of polarization response in the EM field. According to the effective medium theory, the ε′ value is related to the sample conductivity. In general, the higher conductivity leads to a higher ε′ value, but too high ε′ tends to generate an eddy current on the sample surface, resulting in the reflection of EM waves. This is unbeneficial to EM wave absorption and thus might worsen impedance matching. The ε′ value between 4 and 5 is the best according to the previous report [47]. It can be found from Figure 8a that the ε′ value of HEO-Cu is very close to this criterion, indicating the best impedance matching of HEO-Cu and the worst of HEO-Zn. That the largest ε′ value of HEO-Cu ferrite throughout the range of 2–18 GHz is possibly due to the fact that the conductivity of Cu ranks only second to Ag, thereby Cu addition can increase the dielectric loss. Moreover, it can be seen that the imaginary part of permittivity (ε″) of HEO-Cu still displays a leading trend among the three samples, indicating that the Cu−containing ferrite not only achieves high dielectric storage but also exhibits enhanced dielectric loss. It is noteworthy that the ε″ value of HEO-Cu drops sharply at the range of 2–6 GHz and shows a repeated increasing−decreasing trend at 6–18 GHz. The decrease at low frequency is probably due to the 3D porous structure, which can significantly increase the volume fraction and thus optimize the impedance matching. The relatively stable trend at high frequency is likely owing to the synergistic effect of space charge, interface and orientation polarization, resulting from the large specific surface area and heterogeneous chemical constitution of HE spinel ferrites. The dielectric loss tangents (tan*δ*_ε_) of the three samples are shown in Figure 8c, which have a similar variation trend with ε″ value. The tangent depicts the dielectric loss capability for the entered electromagnetic waves, and these curves possess multiple resonance peaks, indicating the presence of polarization loss [48,49,50,51,52]. 

According to Debye’s theory, the *ε*′ and *ε*″ values can be expressed as:

(2) $$\varepsilon^{\prime }=\varepsilon_{\infty }+\frac{\varepsilon_{s}-\varepsilon_{\infty }}{1+{\left(2\pi f\right)}^{2}r^{2}}$$

(3) $$\varepsilon^{\prime \prime }=\frac{\varepsilon_{s}-\varepsilon_{\infty }}{1+{\left(2\pi f\right)}^{2}r^{2}}\omega \tau$$

where *ε_s_* and *ε*_∞_ are the static and optical dielectric constants, respectively, *ω* (*ω* = 2*πf*), *f*, and *τ* represent the angular frequency, frequency, and polarization relaxation time, respectively. Moreover, the Cole–Cole equation is derived from (2) and (3):

(4) $$(\varepsilon^{\prime }-\frac{\varepsilon_{s}-\varepsilon_{\infty }}{2}{)}^{2}+({\varepsilon^{\prime \prime })}^{2}=(\frac{\varepsilon_{s}-\varepsilon_{\infty }}{2}{)}^{2}$$

**Figure 9:**
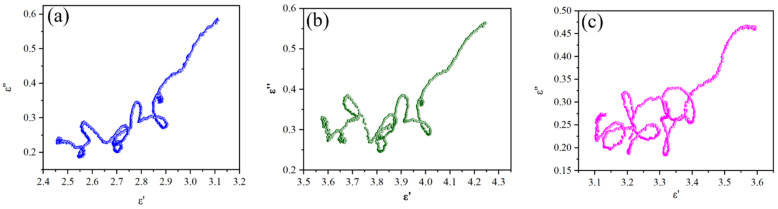
Cole–Cole plots of (**a**) HEO-Zn, (**b**) HEO-Cu, and (**c**) HEO-Mn.

In order to further analyze the dielectric loss mechanism, the Debye relaxation was analyzed by the Cole–Cole curve, as shown in Figure 9. Debye relaxation is an important loss mechanism for the dielectric loss of absorbers. In Cole–Cole curves, each semicircle represents a polarization relaxation process. It is seen that each Cole–Cole curve exhibits several obvious semicircles, which also present serious distortion, indicating that the polarization effect plays a leading role in the dielectric loss. 

**Figure 10:**
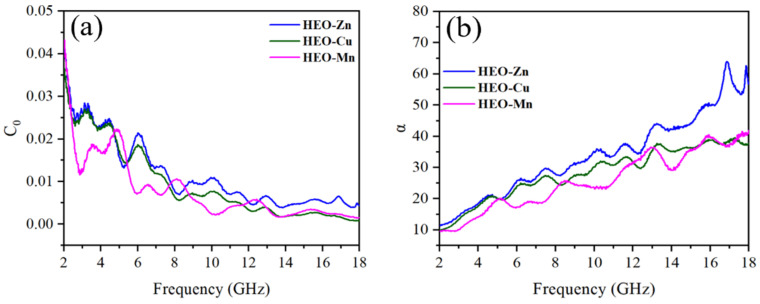
The relationship of (**a**) C_0_ and (**b**) α with frequency of HEO-Zn, HEO-Cu, and HEO-Mn.

Magnetic loss is primarily estimated by magnetic permeability [53]. Figure 8d,e shows the real part (*µ*′) and imaginary part (*µ*″) of the permeability of the three ferrites, respectively. The dramatic fluctuations are observed in the *µ*′ and *µ*″ curves, manifesting the significant magnetic loss. Notably, the *µ*′ value of HEO-Cu is relatively small mainly due to the high conductivity of Cu. The internal magnetic field generated by the weak induced current would resist the external magnetic field, thus generating the radiated magnetic energy and suppressing magnetic loss. Hence, the high conductivity usually leads to a small *µ*′. The *µ*″ values are generally related to *H*c, and the larger *H*c can give the absorber higher *µ*″. It can be seen that HEO-Zn has the largest *µ*″ value, HEO-Mn the lowest, and HEO-Cu in the middle, in accordance with the results of *H*c analyses above. The magnetic loss tangents (tan*δ*_µ_) shown in Figure 8f can be used to evaluate the magnetic loss ability of the absorber for the entered electromagnetic waves. The order of tan*δ*_µ_ values within the whole frequency from high to low is HEO-Zn, HEO-Cu, and HEO-Mn, which is basically consistent with their magnetic properties (see Figure 5 and Table 1). In addition, the type of magnetic loss can be analyzed by the parameter of *C*_0_ with the expression:

(5) $$C_{0}=\mu^{\prime \prime }{\left(\mu^{\prime }\right)}^{-2}\mu^{\prime \prime }{\left(f\right)}^{-1}=2\pi \mu_{0}d^{2}\delta$$

where *d* is the thickness of the specimen, *f* is the frequency and μ is the vacuum permeability. Figure 10a shows the relationship between *C*_0_ and the frequency of HEO-Zn, HEO-Cu, and HEO-Mn samples. It can be seen that the *C*_0_ curves are disordered and unstable, indicating that the eddy current loss is not the main loss mechanism in the magnetic loss. Furthermore, these *C*_0_ curves possess many distinct resonance peaks, which are primarily associated with natural resonance loss [54]. 

The attenuation constant of α describing the EM energy attenuation capability of the absorber can be denoted as the Formula (6):

(6) $$\alpha =\left(\sqrt{2}\pi f/c\right)\sqrt{\left(\mu^{\prime \prime }\varepsilon^{\prime \prime }-\mu^{\prime }\varepsilon^{\prime }\right)+\sqrt{{\left(\mu^{\prime \prime }\varepsilon^{\prime \prime }-\mu^{\prime }\varepsilon^{\prime }\right)}^{2}+{\left(\mu^{\prime }\varepsilon^{\prime \prime }+\mu^{\prime \prime }\varepsilon^{\prime }\right)}^{2}}}$$

where all the characters have been explained hereinabove. The relationship between α and frequency is shown in Figure 10b. Although HEO-Zn has the largest attenuation constant within the whole frequency range, its absorption performance is not satisfactory due to the poor impedance matching (see Figure 8a). Moreover, at the frequency range of 2–8 GHz, the α of HEO-Cu is almost the same as that of HEO-Zn. Moreover, at the range of 8–18 GHz, the α of HEO-Cu is slightly lower than that of HEO-Zn but higher than that of HEO-Mn. Therefore, the best impedance matching and proper EM energy attenuation ability result in the excellent absorption performance of HEO-Cu [55]. 

**Figure 11:**
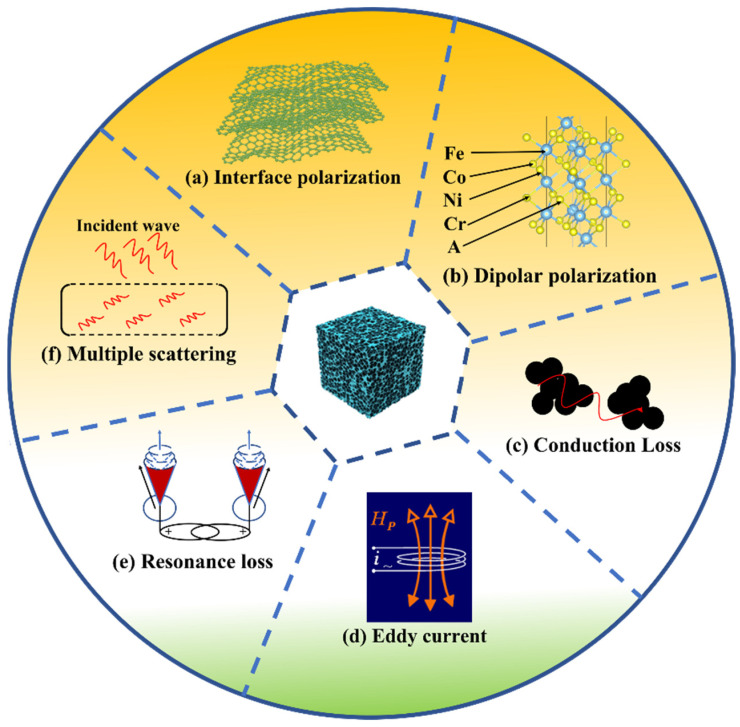
Schematic drawing of EM wave absorption mechanism of the as-prepared HE spinel ferrites.

The relevant EM wave absorption mechanism of HEO-Cu ferrite is shown in Figure 11. On one hand, for dielectric energy loss, the porous structure and proper conductivity of the HEO-Cu sample effectively modulate the dielectric constants, ensuring more EM waves enter the absorber. That is to say, the charge can accumulate a large number of sample-air interfaces due to the difference in conductivity, leading to interfacial polarization [56,57]. Besides, the dipoles in the spinel structure are reoriented under the effect of the applied electromagnetic field, which favors dipolar polarization. Furthermore, the combination of the variable valence states of Fe, Co, Ni and Cr elements, low permeation threshold of Cu (0.24% volume percentage in Cu nanoparticle), high electrical conductivity, and 3D conductive network can improve the conduction loss for dielectric energy decay. On the other hand, due to the excellent magnetic properties of HEO-Cu, the electromagnetic energy in the EM field can be effectively attenuated by the eddy current and natural resonance effects between magnetically coupled interfaces. Basically, compared with HEO-Zn and HEO-Mn, HEO-Cu possesses much better EM absorption properties. This is mainly because HEO-Cu has a higher dielectric constant (Figure 8a), indicating the enhanced dielectric loss behavior, which is believed to play a dominant role in the EM absorption and loss process. Furthermore, the HEO-Cu sample has a residual CuO phase, which can not only increase the consecutiveness and transmission of electrons but also provide abundant interfaces that may improve the interfacial polarization. Besides, Cu element is a diamagnetic substance [58], which would strengthen the internal magnetic field rather than shield the external magnetic field, thus increasing the magnetic loss. 

The microstructure of wave-absorbing materials also plays a key role in the absorbing properties. In the present work, the 3D porous structure not only reduces the density of EMAs but also exhibits multiple advantages for improving wave absorption performances [59]. Firstly, the porous structure can reduce the dielectric constants, which is conducive to optimizing the impedance matching and thus enables more EM waves into the absorber. Soon afterward, the EMA could absorb more EM waves instead of reflection, due to the repeated reflecting and scattering of the incident microwaves in the internal empty pores. Secondly, the polarization centers, pores or cracks are beneficial to space charge polarization. Hence, in a porous structure, the remaining air can be used as the effective medium to produce significant relaxation loss in alternating EM field [60]. Finally, the porous structure with multitudinous chemical constituents provides the 3D conductive network and has a positive effect on broadening the absorption band. Most importantly, the present HE spinel ferrites show high structure thermostability, and thus charge carriers could be motivated at high temperatures, thereby benefitting the wave-absorbing process. Therefore, the porous HE spinel ferrite seems to be a good candidate to serve as a high-temperature wave-absorbing material. 

## 3. Material and Methods 

### 3.1. Raw Materials and Reagents

The raw materials of Fe_2_O_3_ (Ferric sesquioxide), Co_3_O_4_ (Cobalt (II, III) oxide), NiO (Nickel oxide), Cr_2_O_3_ (Chromium sesquioxide), ZnO (Zinc oxide), CuO (Cupric oxide), MnO (Manganese (II) oxide) were purchased from Sinopharm Chemical Reagent Co., Ltd. (Shanghai, China). CH_3_CH_2_OH (Anhydrous ethanol) was purchased from Aladdin Biochemical Technology Co. Ltd. (Shanghai, China). All chemical reagents used in this work were of analytical grade, and deionized water was used throughout this study. 

### 3.2. Preparation Process

**Figure 12:**
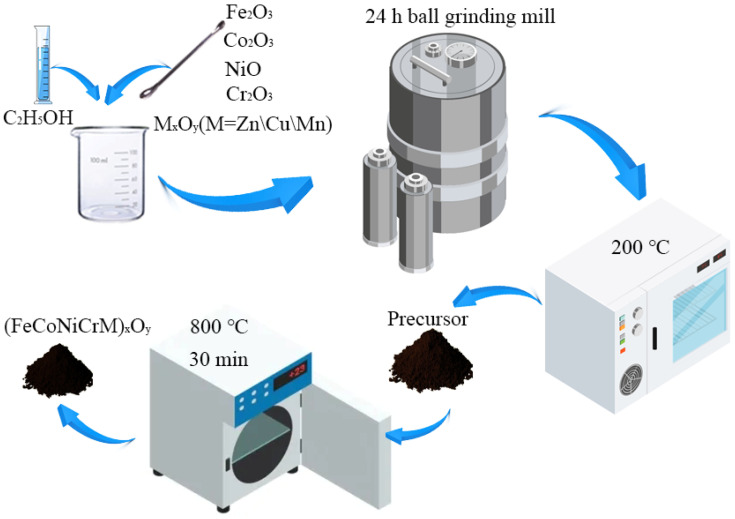
Schematic illustration of the preparation of (FeCoNiCrM)_x_O_y_ (M = Zn, Cu, and Mn).

All the ferrites of (FeCoNiCrM)_x_O_y_ (M = Zn, Cu, and Mn) were prepared by solid-state reaction. Taking (FeCoNiCrMn)_x_O_y_ as an example, the synthesis process is described in detail as follows, which is also presented in the schematic drawing of Figure 12. Firstly, the oxides of 0.03 M Fe_2_O_3_, 0.06 M NiO, 0.02 M Co_3_O_4_, 0.03 M Cr_2_O_3_, and 0.06 M MnO were mixed with 50 mL anhydrous ethanol and then put into a mill pot. The ball-milling proceeded for 24 h in order to obtain the homogeneous oxide mixture. Subsequently, the mixture was dried in an oven and ground for 10 min to get black powders. Finally, the powders were heated to 800 °C in a furnace for 30 min, and afterward, the powders were taken out from the furnace and repeatedly ground for 10 min. 

### 3.3. Characterization

The structures of as-prepared ferrites were conducted by X-ray diffraction (XRD) with Cu Kα radiation. The microstructure analyses were characterized by field-emission scanning electron microscopy (FSEM, Apreo S HiVac), coupled with energy-dispersive X-ray spectroscopy (EDS). The valence state and binding energy were determined by X-ray photoelectron spectroscopy (XPS, AXIS SUPRA+). The magnetic properties were measured by Vibrating Sample Magnetometer (VSM, Oxford 1 Tesla). The thermostability was analyzed by TG curves, performed by STA 449C Jupiter differential scanning calorimeter (DSC) at a heating rate of 10 K/min, through the services from Sci-go Instrument Testing Platform. 

### 3.4. EM Parameter Measurement

The microwave absorbing properties were analyzed by a Vector Network Analyzer (VNA, Advantest type R3770) in the frequency range of 2.0–18.0 GHz. After mixing with paraffin, the HE spinel ferrites were pressed into a ring tube. The height, outer diameter, and inner diameter of the pipe are approximately 2.00 mm, 7.00 mm and 3.04 mm, respectively. The mass ratio of ferrite powders to paraffin is about 1:1. Based on the coaxial method, EM parameters (*ε_r_* and *μ_r_*) of the mixture were obtained on a vector network analyzer (VNA, Agilent N5222A). The RL values were calculated according to Formulas (7) and (8) [56]:

(7) $$Z_{in}=Z_{0}\sqrt{\mu_{r}/\varepsilon_{r}}\tan h\left[j\left(2\pi fd/c\right)\sqrt{\mu_{r}\varepsilon_{r}}\right]$$

(8) $$RL\left(dB\right)=20\log\left|\left(Z_{in}-Z_{0}\right)/\left(Z_{in}+Z_{0}\right)\right|$$

where *ε_r_* and *μ_r_* are the complex permittivity and permeability, *d* is the thickness of the sample, *f* is the frequency, *c* is the speed of light in a vacuum, and *Z*_0_ and *Z_in_* are the free space impedance and input impedance, respectively. 

## 4. Conclusions

In this work, HE spinel ferrite powders of (FeCoNiCrM)_x_O_y_ (M = Zn, Cu, and Mn) were synthesized by the simple solid-phase reaction at 800 °C for 30 min. These HE ferrite powders exhibit the uniform distribution of chemical components with almost the same atomic mole proportion and homogeneous 3D submicron porous structure with a pore diameter in a range of tens to hundreds of nanometers. The TG measurements show that the present HE spinel ferrites have ultrahigh structural stability although increasing the temperature up to 800 °C, the mass change is still very small (less than 1.0%). The performance measurements show that the present HE spinel ferrites possess considerable RL_min_ and significantly enhanced EAB. The RL_min_ and EAB values of HEO-Zn and HEO-Mn samples are about −27.8 dB at 15.7 GHz, 6.8 GHz, and −25.5 dB at 12.9 GHz, 6.9 GHz, with a matched thickness of 8.6 and 9.8 mm, respectively. Especially, the RL_min_ value of HEO-Cu is −27.3 dB at 13.3 GHz with a matched thickness of 9.1 mm, and notably, the EAB reaches about 7.5 GHz (10.5–18.0 GHz), which covers almost the whole X-band range. The mechanism analyses indicate that the superior absorbing properties of HEO-Cu are primarily attributed to the dielectric energy loss involving interface polarization and dipolar polarization, and the magnetic energy loss referring to eddy current and natural resonance loss. Besides, the porous structure can optimize the impedance matching, strengthen space charge polarization, and provide a 3D conductive network, thus benefiting the wave absorption performances. These results manifest the potential application prospect of the porous HE spinel ferrites in the EM wave absorption field. 

## Figures and Tables

**Table 1:** The magnetic properties of Ms, Hc, and Br of HEO-Zn, HEO-Cu, and HEO-Mn.

Samples	Ms /emu/g	Hc /Oe	Br /emu/g
HEO-Zn	12.0	433.4	5.5
HEO-Cu	8.2	278.6	3.9
HEO-Mn	0.48	0.1	0.1

**Table 2:** The wave absorbing properties including RL_min_, matching thickness, and EAB values of the present three ferrite samples, as well as many reported EM absorbing materials.

Samples	RL_min_ /dB	Thickness /mm	Frequency /GHz	Ratios /wt%	EAB /GHz	Ref.
RGO/Ti_3_C_2_T_x_ hybrids	−22.0	3.6	-	-	4.0	[42]
CoFe@Ti_3_C_2_T_x_	−36.3	2.2	-	60.0	2.6	[43]
M-Ti_3_C_2_T_x_/Ni	−24.3	2.2	-	60.0	2.6	[44]
M-Ti_3_C_2_T_x_/ZnO	−26.3	4.0	-	25.0	1.4	[45]
FeCrMoNiPBCSi	−23.1	6.6	4.2	20.0	2.2	[3]
Ti_3_C_2_ nanosheets filled composites	−11.0	1.4	-	-	5.6	[46]
Ti_3_C_2_T_x_	−17.0	1.4	-	-	5.6	[46]
HEO-Zn	−27.9	8.6	15.7	50.0	6.8	This work
HEO-Cu	−27.3	9.1	13.3	50.0	7.5	This work
HEO-Mn	−25.5	9.8	12.9	50.0	6.9	This work

## Data Availability

No new data were created or analyzed in this study. Data sharing is not applicable to this article.

## Sample Availability

Samples of the compounds are available from the authors.
